# A Bias-Aware Analytic Framework for Real-World Data with AI-Audited Workflows: Application to Opioid Ordering in Pediatric Oncology

**DOI:** 10.3390/bioengineering13101098

**Published:** 2026-09-23

**Authors:** Tricia Morphew, Hannah Pease, Michelle A. Fortier, Zeev N. Kain, Lois W. Sayrs

**Affiliations:** 1 Rady Children’s Health, 1201 W La Veta Ave, Orange, CA 92868, USA; tricia.morphew@choc.org or tricia@morphewconsulting.com or tmorphe1@jh.edu (T.M.); michelle.fortier@choc.org (M.A.F.); zkain@hs.uci.edu (Z.N.K.); 2 Johns Hopkins Bloomberg School of Public Health, Johns Hopkins University, 615 N Wolfe St, Baltimore, MD 21205, USA; 3 Joe C. Wen School of Population & Public Health, University of California, Irvine, 856 Health Sciences Rd, Irvine, CA 92617, USA; peaseh@uci.edu; 4 Center on Stress & Health, University of California, Irvine, Irvine, CA 92617, USA; 5 Department of Anesthesiology and Perioperative Care, University of California, Irvine, Irvine, CA 92617, USA; 6 Sue & Bill Gross School of Nursing, University of California, Irvine, Irvine, CA 92617, USA; 7 Department of Pediatrics, School of Medicine, University of California, Irvine, 1001 Health Sciences Rd, Irvine, CA 92617, USA

**Keywords:** real-world data (RWD), large language models (LLMs), artificial intelligence (AI), bias (epidemiology), linear modeling, pediatrics, medical oncology

## Abstract

**Background:** The growing use of computational modeling of real-world data (RWD) in clinical research introduces significant risks for data scientists and AI trainers already managing inconsistency, unexamined bias, and analytical opacity. Building upon more than 600 guideline-based checklists and pre-planning templates for data quality and transparency reporting in observational RWD, we propose a run-time operational execution loop to embed reproducibility analytics within the workflow. Our approach acts as a run-time gatekeeper mapping prespecified analytics goals to a closed-loop LLM semantic code auditor, checking for both logic, bias, and vulnerabilities during code execution. Our framework was designed to bridge the gap between passive disclosure, ensuring proposed requirements, and *post-hoc* validation. Our structured hierarchical approach coupled with AI auditing is the first phase in the development of full-stack automation of bias-aware RWD analytic validation. **Methods:** Our analytic framework spans 10 domains across three analytic phases. Each domain includes prespecified criteria governing verification with potential biases, mechanisms, and mitigation strategies systematically mapped throughout. Phase I entails specification (Domains 1–4: objectives and estimands, data inputs and outputs, cohort and filters, and variable coding); Phase II: modeling (Domains 5–7: model testing, diagnostics, and reasonableness checks); and Phase III: robustness and interpretation (Domains 8–10: missing data handling, temporal and stratified checks, and scope boundaries). We utilize specific prompt-directed large language model (LLM) auditing of model elements to ensure data quality. The methodology is illustrated with examples from an empirical characterization of inpatient opioid data from the Cerner Real-World Data (2014–2021). GLMMs and corresponding outputs were generated in R v4.4.2. Curated AI-auditing of first-pass code drafting was performed using ChatGPT 5.2 (OpenAI), with cross-LLM portability assessed using Claude Sonnet 5 (Anthropic). **Results:** The Cerner RWD database contained 16,734 pediatric cancer patients (76,395 encounters) across 55 U.S. health systems. During the specification phase, total daily dose could not be estimated with sufficient validity from the Cerner RWD, and “as-needed” (PRN) orders, warranted empirically supported exclusion. The modeling phase revealed residual variability in opioid ordering attributable primarily to patient-level differences within health systems (ICC = 19.9%) rather than between health systems (ICC = 2.0%). Temporal analysis revealed divergent opioid-ordering trajectories from 2017 onward, with decreasing morphine and increasing fentanyl ordering. We then utilized curated prompting to enforce an AI-generated coding requirement to produce valid likelihood ratio tests across the prespecified model sequence accounting for temporal trends. After harmonizing the model specification and estimation settings, both LLM workflows produced identical random-effect variance estimates, ICCs, and AIC values evaluated through the third decimal place, with corresponding fixed-effect estimates likewise matching. **Conclusions:** The 10-domain framework provides a structured, reproducible mechanism for governing RWD analyses by using a closed-loop semantic code auditor to embed run-time analytics directly into the data science workflow, supporting transparent exposure definition, systematic bias assessment, and prespecified analytic decision-making. By bridging the gap between passive transparency checklists and active automation, the framework operationalizes existing data quality guidelines through an executable, bias-aware code-auditing process. Cross-LLM concordance in identifying problematic logic and reproducing complex workflows supports the portability of this run-time gatekeeper. Ultimately, this approach establishes a scalable pathway for embedding expert statistical decision-making into the automation of safe, reliable real-world evidence (RWE) generation.

## 1. Introduction

The growing use of computational modeling of real-world data (RWD) in clinical research introduces significant risks for data scientists and AI trainers already managing inconsistency, unexamined bias, and analytical opacity. The risks are substantial with the democratization of large administrative databases where there is little-to-no technical barrier to access, although general access may be subject to legal constraints on use. Clinical studies using RWD routinely rely on clinical or administrative data generated outside controlled research settings, and results increasingly depend on derived analytic outputs from unaudited data and analytics. Seemingly simple measurements, e.g., weight-based pharmacological metrics in pediatric clinical research, are a good example of a confounded, often unreliable estimate of dose. In constructing an emulated trial or digital twin from RWE in a quasi-experimental dose-escalation emulated trial, the unreliability of one simple measure could invalidate the research. Real-world evidence (RWE) requires careful specification, a sensitivity to the need for hierarchical modeling, and iterative validation to ensure reliability and reproducibility [1,2]. In pediatrics, these challenges are compounded by well-documented data quality limitations, including high missingness and inaccuracy in weight data [3], fragmented medication capture across EMR and dispensing systems, and unknowingly censored data extractions that seriously circumscribe researchers’ ability to self-discover data limitations [4].

This example highlights the hidden risks within universal access to data via platforms like EPIC Research or EPIC Cosmos, as well as the increasing reliance on LLMs for population and pattern discovery. While more open access has the potential to broaden analytic accessibility, reliance on requirement-driven data extractions (and more frequently, LLM-generated code) can easily introduce increased risks of inconsistency, unexamined bias, and analytical opacity that can undermine standards for RWD evidence generation [5,6,7,8,9].

While there are more than 600 guideline-based checklists and pre-processing templates that exist for data-quality assessment and transparency reporting across study designs, clinical domains, and report sections [10,11,12,13,14,15,16,17], including the NICE Real-World Evidence Framework and its accompanying Data Suitability Assessment Tool (DataSAT) [18], guidelines alone cannot ensure reproducibility. Ongoing initiatives to address comparability across data systems, including initiatives to standardize data elements across U.S. healthcare systems and leverage digital twinning, represent meaningful progress [19,20]. However, standardizing confounded data do not address the underlying invalidity, nor do they address the challenges of working with non-standardized data. Consequently, critical decisions regarding bias mitigation, specification errors, data missingness, robustness, and verification remain highly vulnerable to ad hoc human judgment. The advent of large language models (LLMs) creates an opportunity to operationalize expert statistical guardrails directly within the live execution workflow, bounding analytical adjustments and mitigating systematic bias.

Aligning study objectives with a reproducible analytic workflow remains a persistent challenge in clinical research using RWD, as published analyses rarely disclose the precise pre-processing and modeling choices that most affect data integrity and reproducibility [21]. The growing use of LLMs to assist with or generate analytic code introduces additional complexity; without structured oversight, LLM outputs can be inconsistent or inaccurate, underscoring the need for reasoning accuracy [22] and systematic task auditing [5,6,7,8,20,23]. To address these challenges, we propose a bias-aware, 10-domain analytic framework that transitions data quality assurance from retrospective disclosure to active, run-time governance. By embedding a closed-loop semantic auditor directly into the run-time execution environment, this approach shifts the paradigm from passive checklists to live workflow intervention. Our system evaluates code dynamically as it runs, mapping prespecified analytics goals against the executing script to flag logical contradictions, specification errors, and systematic biases before outputs are generated.

Spanning three core phases—specification, modeling, and robustness/interpretation—this framework transforms the role of large language models (LLMs) from a passive coding assistant to a conservative, self-auditing run-time monitor. We demonstrate its utility using an empirical characterization of inpatient opioid ordering among pediatric cancer patients within the Cerner RWD database—an environment where explicit data limitations are not well described [24,25,26]. Not meant as a critique of the database, this example illustrates how our run-time operational loop systematically identifies and mitigates data constraints inherent to any RWD ecosystem.

While full automation of this process remains a future goal, this framework establishes the foundational mechanics required to achieve it. The system is designed to ingest and formalize expert statistical guidance, embedding advanced biostatistical rulesets directly into the prompt architectures that drive the auditor. By codifying human expertise to actively guide the AI-driven runtime engine, this methodology provides biostatisticians, data scientists, AI trainers, and regulatory stakeholders with a scalable, executable pathway toward a future of fully automated, expert-driven, and run-time audited health data analytics.

## 2. Methods

### 2.1. Analytic Framework and Audited Workflow

The analytic framework is organized across ten discrete domains grouped into three sequential, iterative phases (Figure 1). This iterative design ensures that runtime analytics can continually inform and refine earlier stages of the workflow as data constraints are uncovered.

The analytic framework (Figure 1) is organized into three phases that can be used sequentially but are more effective if utilized iteratively to ensure comprehensive assessment: (Phase I) specification (Domains 1–4)—objectives/estimands, data inputs/outputs, cohort/filters, and variable coding; (Phase II) hierarchical modeling (Domains 5–7)—model specification, diagnostics, and reasonableness checks; and (Phase III)—robustness and interpretation (Domains 8–10)—missing data handling, temporal/stratified checks, and scope boundaries.

Descriptive analyses and data transposition were performed in IBM SPSS Statistics v29.0. To verify results, we constructed an audit process utilizing a curated AI workflow that targeted the three phases of the analytic framework, (specification, hierarchical modeling, and robustness and interpretation). For each phase and domain, we constructed and tested curated, structured prompt templates. We also developed verification criteria, and interpretation guidance is summarized in Table 1. AI-audited first-pass code drafting for R version 4.4.2 was performed using Chat GPT-5.2 (OpenAI) in a privacy-preserving local environment, with cross-LLM portability and diagnostic verification assessed using Claude Sonnet 5 (Anthropic); no patient-level data were submitted to the LLM. Final model execution, diagnostic validation, and clinical interpretation remained strictly under analyst and biostatistical oversight.

Domains are iterative, not strictly sequential; return to earlier domains when execution errors, diagnostic failures, or reasonableness concerns are identified. In this exemplar, some safeguards were added only after first-pass model execution and expert review and therefore should not be interpreted as having been prespecified a priori. LLMs were used to translate structured analytic specifications into executable code, support iterative refinement, and incorporate expert-defined verification checks, safeguards, and alternative modeling pathways into subsequent workflow iterations. Automated checks identified conditions warranting review but did not replace methodological judgment; approved decisions could then be encoded into revised AI-assisted code and re-verified. Model execution, methodological adjudication, diagnostic review, and interpretation remained investigator-led in a secure local environment without sharing clinical data.

### 2.2. Phase I. Specification (Domains 1–4)

General run-time auditing procedures for Specification (Domains 1–4)—Prompts were structured to generate R code for estimating encounter-level opioid order probability with prespecified effect and variance estimands. Co-primary estimands were specified a priori: adjusted odds ratios (aORs) for encounter-level opioid ordering across prespecified covariates, and cluster-level variance components and ICCs for each level of the prespecified hierarchy. Prompt inputs included the local data import path; output artifact requirements including destination directory, filename prefix, and traceable export naming conventions; variable definitions specifying continuous versus factor classification with prespecified reference levels; and cohort and filter validation checks against expected sample size counts at each sequential filter step. Verification prior to modeling confirmed that output files were generated with traceable filenames, input/output counts and variable types were validated against the data dictionary, analytic N reconciled with the cohort flow at each filter step, and distributional checks and outlier identification were completed before modeling proceeded.

Outputs and prompt refinement—Outputs were required to meet all prespecified verification criteria prior to interpretation, as specified in Table 1. These criteria were particularly important given the data quality limitations documented in Domains 3 and 4, including fragmented medication capture and high missingness in dose elements. When execution errors occurred, error-informed prompt refinement was performed by rerunning revised code until all verification criteria were met; illustrating the iterative, bias-aware auditing process the framework is designed to support. One such refinement was required when 308 records with missing sex were inadvertently included in M1–M2, preventing valid likelihood ratio tests for nested model comparisons; as noted below in Phase III, code was updated to enforce a common complete-case sample across all models. Because missingness was less than 1%, imputation and additional sensitivity analyses were not warranted.

#### 2.2.1. Domain 1. Identify and Confirm Research Objective and Estimands

Domain 1 aligns the study objectives with the underlying data architecture, ensuring that target estimands are supported within the database architecture. Like many health-related databases, Cerner primarily captures clinical billing data tied to unique patient encounters, mapping various medications, procedures, and treatments. Our analytical workflow in this illustration was driven by the primary study objective: to evaluate and compare opioid prescribing patterns across pediatric cancer patients while accounting for heterogeneous cancer diagnoses, demographics, and clinical characteristics that may influence cumulative dosing. Statistical guidance was codified *a priori* to define specific primary and secondary estimands, specifically: (1) encounter-level outcomes across prespecified covariates; (2) patient-level variance components; and (3) cluster-level variance components; (4) time-related variance components. To ensure comparability across the patient cohort, the target metric was defined as morphine milligram equivalents (MMEs) calculated from weight-based opioid dosing (mg/kg). The primary unit of analysis was the clinical encounter, with the patient serving as the secondary unit. Because these multi-institutional data originate at the hospital level, institutional variation and shifting longitudinal prescribing guidelines were systematically integrated into the Domain 1 structural assessment.

#### 2.2.2. Domain 2. Data Inputs and Outputs

Inputs−Patient characteristics, health-system characteristics, and opioid-order data were the primary inputs and were extracted from the Cerner RWD in August 2023. Candidate predictors, operational definitions, analytic parameterizations, and intended uses are summarized in Table 2. Feature parameterization was guided by a combination of published guidelines, clinical reasoning, the prior literature, and the observed distribution of each variable. To guarantee transparency and reproducibility, these features were prespecified within the data pre-processing pipelines and the underlying analytic code.

For each patient, we extracted the first recorded cancer diagnosis which was classified by eight clinically derived categories using ICD-10 codes C00–C96 or comparable ICD-9 codes. Current pediatric clinical guidelines, empiric institutional practices, and maximum inpatient pain scores were identified as primary inputs to MMEs. Initial descriptive analyses of the pain input was performed based on the scale score for the pain metric (0–10) with the expectation that the input would be bounded by pediatric guidelines for opioid administration, with variation driven within and between institutions and/or hospital departments (oncology vs. PICU) based on empiric practice. However, run-time evaluation revealed substantial inconsistencies in how pain scores are recorded across hospital days. Unmeasured recording biases were evident, and approximately 25% of encounters lacked pain data. These data-quality limitations triggered an automated runtime assessment within our framework to evaluate whether the metric should be excluded or imputed. If imputed, the framework would require explicit algorithmic management to bound and minimize the impact of this unmeasured bias. For the present study, pain was therefore excluded from the modelling procedure. The primary exposure estimand was constructed from inpatient opioid data, defined as binary encounter-level exposure (one or more opioid orders during the encounter versus none). The formal exposure window and operational architecture are detailed in Domain 4. Prior to model execution, eligibility criteria and cohort filters were prespecified to ensure all processing steps were locked to the target study population.

Outputs—To enforce structural reproducibility, output directories, file prefixes, and precise naming conventions were mapped *a priori*. This structure ensured that all exported data validation checks, tables, figures, model objects, and fit statistics could be directly traced back to their exact analytic origin. Distributional summaries and model outputs were systematically generated in standardized formats optimized for documentation. Automated runtime flags were deployed within the execution loop to dynamically isolate data anomalies or model convergence warnings. However, the system does not operate blindly; all flagged outputs underwent mandatory expert review to validate automated findings, detect unflagged anomalies, and ensure the clinical and statistical interpretability of the results prior to final reporting.

#### 2.2.3. Domain 3. Cohort and Filters

Inclusion and exclusion criteria—These criteria are described in Figure 2. To reduce confounding and selection bias, we excluded encounters with missing (n = 50) or invalid (n = 34) length of stay (LOS), encounters classified as Labor and Delivery (n = 6), and health systems with fewer than five cancer-diagnosis encounters (n = 48), the latter supporting more stable assessment of between-system variability. Given the file size, these exclusions were not considered material to inducing censoring. Encounter series were truncated at each patient’s 20th hospitalization, meaning later encounters among high-utilization patients were not included in analyses. This analyst-defined threshold, established *a priori*, corresponded approximately to the 98th percentile of the patient-level encounter count distribution and was selected to limit the influence of a small number of extreme high-utilization observations while supporting computational and statistical stability across hierarchical levels. The audit did not formally evaluate alternative encounter thresholds or prespecify sparsity-based criteria for determining this cut-point. Formal sensitivity analyses were therefore not performed in the present feasibility analysis; the implications of this limitation and opportunities for extending the cohort-definition audit are addressed in the Discussion section. Encounters with an LOS > 30 days were assessed, considered clinically as a distinct prescribing pattern based on prolonged hospitalizations that may reflect distinct prescribing patterns for subacute or chronic pain management, and therefore were excluded (n = 3665). However, this exclusion could be managed as a separate sub-cohort in future analyses. Of note, the >30 day LOS was not randomly distributed across patients; therefore, its potential effect on characterization of opioid ordering among higher-complexity patients would require sub-cohort analysis prior to publication or could be targeted to be addressed in the limitations section of the Discussion section. The final analytic cohort comprised 76,395 encounters from 16,734 patients across 55 geographically and organizationally diverse U.S. health systems. As the unit of analysis is encounters rather than patients, high-utilization patients contribute more observations, which is addressed through the hierarchical modeling framework described in Domains 5–7.

Investigation of illogical encounters—Initial data extraction produced a medication file containing 225,508 opioid prescription orders across all encounter records. To establish a standardized core, the dataset was restricted to the five most common opioid types (morphine, fentanyl, oxycodone, hydromorphone, and methadone), which accounted for 96.9% of all orders (n = 218,260 order rows). The framework’s runtime auditing protocols then applied sequential exclusion criteria to flag and isolate structural anomalies and illogical data entries (see Supplemental Table S1). First, orders with a start date earlier than the patient’s admission date, indicative of left-censoring or data entry error, were excluded (n = 1399). Second, orders explicitly marked with a status of “Cancelled” or “Error Entry Deleted” were removed (n = 2148). Third, to isolate precise drug tracking, orders containing one of the five primary opioids in combination with another active ingredient were excluded (n = 4822).

The framework’s run-time audit revealed a critical platform-specific constraint regarding “As-Needed” (PRN) tracking: completed PRN administrations generated separate, unlinked rows that stripped the as-needed flag, breaking the relational database link between prescription and fulfillment. Consequently, orders with an as-needed status marked as True (n = 86,636) were segregated. To verify this structural constraint during runtime, a closed-loop subset comparison of discontinuation rates between as-needed “True” (n = 86,636) and as-needed “False” (n = 123,255) orders was conducted and findings described in the subsequent measurement bias section. This audit confirmed that the data-logging mechanisms fundamentally altered the observed behavior of PRN fields, validating their exclusion from primary modeling. The remaining 123,255 non-PRN orders comprised the final primary analytic sample for all subsequent validation and modeling steps.

#### 2.2.4. Domain 4. Variable Coding

Drug exposure window and operationalization—For each encounter and each of the five primary opioids, the exposure window was defined from admission through discharge. Any prescribed days’ supply extending beyond discharge was excluded from the encounter-level exposure calculation, and the 0.3% of orders with a missing end date were assumed applicable only on the start date. Five drug-specific binary variables were created to denote whether each order referenced the respective opioid (1 = Yes, 0 = No). Data were then aggregated by encounter, hospital day, and opioid type across all valid orders, with the maximum value retained for each opioid-day, to account for multiple orders of the same drug on a given day.

Three exposure variables were constructed from this aggregated structure: daily exposure, indicating opioid use on a given hospital day; cumulative exposure, measured as opioid-days summed across all opioids over the full hospitalization; and ever exposure, a binary indicator of any opioid use during the encounter. Together, these variables support assessment of individual opioid contributions to overall exposure, PRN prescribing patterns, and variance partitioning across cluster levels including ICC estimates. The ever-exposure outcome at the encounter level serves as the primary analytic outcome for the AI-audited coding exemplar demonstrated in this framework.

Runtime verification of daily opioid dose and MME estimation—Without actual dosing available, we executed an evaluation of the extraction algorithm to estimate daily dose and morphine milligram equivalents (MMEs). This run-time validation process uncovered severe, platform-specific structural constraints that precluded reliable dose calculation.

First, these dose elements (frequency, dose quantity, and administration route) required for calculation reflected the initial prescription order, not the actual administration of the dose, introducing a critical flaw to the structural validity of any calculated MME. Second, there was severe missingness, with complete data for only 35.0% (Supplemental Table S2). Third, multiple medication orders occurring on the same day could have overlapping timestamps that were not reliably distinguishable, precluding accurate reconstruction of individual medication modifications. Although this is a common clinical practice, when clinicians stopped, started or adjusted doses within overlapping time windows, the system logged them as concurrent records rather than a sequential timeline, making it impossible to disaggregate individual order durations to calculate an accurate daily dose. We could not determine reasons for discontinuation or reconcile multiple same-day orders reliably without the clinical guidance of the prescribing physician.

Meta-imputation and non-independence testing—To resolve these data-level gaps, a secondary closed-loop verification effort attempted to associate orders with doses using an external meta-imputation approach. This involved using the Pediatric Health Information System (PHIS) to construct a Gibbs sampler—a Bayesian iterative algorithm—to estimate missing dosages from conditional distributions of patient-level covariates. This approach has been used successfully in genomic imputation [27]. However, because PHIS lacks corresponding patient weight data, this strategy required cross-referencing highly notoriously unreliable pediatric weight estimates from Cerner, introducing severe systematic bias. This imputation strategy was ultimately blocked within our runtime pipeline due to high missingness, preventing the weight-based normalization required for MME conversion. A subsequent multilevel variance partitioning analysis revealed that this missingness was heavily clustered at the health-system level (see Supplemental Figure S1), supporting the contention that incomplete dosage fields reflected institutional documentation policies rather than random missingness. This non-independence mathematically invalidated the use of meta-imputation for multisystem RWD, although such estimations remain feasible within individual, highly standardized health systems going forward.

### 2.3. Phase II. Modeling (Domains 5–7)

Run-time auditing procedures for hierarchical modeling (Domains 5–7)—Model prompts specified logistic GLMMs with a prespecified nested model sequence: M1 (random effects only), M2 (M1 plus system-level factors), M3 (M2 plus patient case-mix), and M4 (M3 + calendar year (fully adjusted)). Verification criteria required that model formulas and covariate blocks matched the prespecified analysis plan, likelihood ratio tests used a common complete-case sample across all models, and all diagnostic flags, including convergence warnings, singularity, and AIC comparisons, were reviewed and resolved or documented before final reporting. A prespecified guardrail prompt was used to enforce consistent estimation methods and flag non-comparable model comparisons before reporting likelihood ratio test results. Crude and adjusted patterns were required to be directionally coherent; discordance between crude and adjusted estimates was prespecified to trigger code or filter review before proceeding to interpretation.

#### 2.3.1. Domain 5. Model Specification

Hierarchal structure and cluster levels—Analyses were conducted within a three-level hierarchical structure to mirror the nested nature of the data: encounters (Level 1) nested within patients (Level 2), and patients nested within health systems (Level 3). This structure was specified within our framework to explicitly account for the non-independence of repeated encounters within individual patients and variation in opioid ordering practices across health systems.

At the health system level, a random effect term was specified to account for unmeasured between-system variation, with fixed effects specified for (1) geographic region (nine U.S. regions), (2) health system volume (three categories based on the interquartile range of median encounters per year: low ≤4, moderate 5–222, and high >222), and (3) temporal trends by admission year (2014–2021). For health systems treating patients across multiple territories, a runtime operational assumption assigned the region with the highest encounter frequency as the system’s primary location. No in silico sensitivity tests were performed evaluating the impact of this assumption during this iteration, representing a clear pathway for follow-up validation.

The framework’s pre-specified domains flagged that the most granular geographic variable available in the Cerner RWD was restricted to the first digit of the patient’s ZIP code. This coarse granularity precluded neighborhood-level evaluation of social determinants of health, exposing a widely acknowledged but unresolvable data-source constraint that the framework successfully surfaced for transparency.

Model building and sequential testing—Hierarchical generalized linear mixed models (GLMMs) were used within our run-time execution pipeline to estimate covariate-adjusted encounter-level opioid order probabilities, drug-specific calendar year effects (adjusted odds ratios [aORs] with 95% CIs), and intraclass correlation coefficients (ICCs). Model building followed a prespecified nested sequence: M1 (random effects only), M2 (system-level covariates added), M3 (system-level plus case-mix covariates), and M4 (fully adjusted: system-level, case-mix, and calendar year).

Model fit was audited at each step using the Akaike Information Criterion (AIC). Prespecified diagnostics included residual checks, influence assessments, and singularity assessment. Because the framework detected negligible variance at the individual encounter level, the encounter-level random intercept was dropped from the final M4 specification. Primary inference focused on ICCs and calendar-year drug-type trends; the nested sequence was therefore executed on the overall opioid order outcome, after which drug-specific analyses were conducted using the final M4 architecture. ICCs from models M1-M4 are presented in Supplemental Table S4. Marginalized probability estimates and adjusted ORs with 95% CIs for the overall outcome and for each drug type across calendar years are presented in Table 3A,B. Covariate-specific aORs for system-level/case-mix fixed effects and complex interaction analyses were outside the scope of this baseline evaluation and are reserved for future work.

Sparse data and algorithmic adjustments—For methadone and hydromorphone, the framework’s automated diagnostic checks triggered pre-specified instability criteria for frequentist GLMMs under sparse data conditions, exposing quasi-separation and convergence failures. Based on these data conditions, the closed-loop architecture triggered switching from a frequentist approach to Bayesian hierarchical models for these two specific drugs, which are summarized using posterior estimates and 95% credible intervals.

#### 2.3.2. Domain 6. Model Diagnostics

Fit and stability—Diagnostic thresholds were prespecified prior to model estimation to ensure objective assessment of model fit and stability. Model fit was evaluated sequentially using AIC comparisons across the nested model architecture, with residual and influence diagnostics reviewed at each step. Convergence warnings and variance component behavior were systematically assessed across model specifications.

A primary pre-specified diagnostic criterion was the evaluation of singularity. Singularity occurs when random effect variance estimates approach zero due to insufficient within-cluster variation or sparse data within cluster levels, indicating that model complexity exceeds what the data can reliably support. Where instability, convergence failures, or singularity were detected, as in the frequentist estimation of methadone and hydromorphone under sparse data conditions, the closed-loop framework blocked further frequentist execution. Alternative model specifications were evaluated and documented, automatically triggering the pre-specified transition to Bayesian hierarchical models for these sparse data structures.

#### 2.3.3. Domain 7: Reasonableness Checks

Coherence and shrinkage—Prior to final model estimation, expected directions and magnitudes of key associations were prespecified based on clinical reasoning and the existing literature. Crude and adjusted estimates were compared to assess coherence; specifically, whether covariate adjustment produced directionally consistent and clinically plausible changes in effect estimates. Hierarchical shrinkage of health system-level random effects was examined to verify that between-system variability was appropriately attenuated under the multilevel structure, consistent with expected behavior under hierarchical modeling. Estimates deviating substantially from prespecified expectations were flagged for review and documented as part of the bias-aware analytic workflow.

Primary Outcome Assessment—Opioids were ordered in 33.5% of 76,087 pediatric cancer encounters (Table 3A). Estimates were derived from the fully adjusted GLMM with random intercepts at the health system and patient levels; the encounter-within-patient random effect was omitted for parsimony given negligible variance in M4, and no singularities were observed for the overall opioid ordering outcome. The final reduced model substantially improved fit relative to the random-effects-only baseline (AIC: 89,671 vs. 92,345; ΔAIC = −2674). Encounter-level opioid ordering outcomes were estimated using frequentist GLMMs with 95% CIs for the overall outcome and for morphine-, fentanyl-, and oxycodone-specific outcomes. For hydromorphone and methadone, sparse events led to convergence warnings and separation-related coefficient instability in frequentist models; these outcomes were therefore refit using Bayesian multilevel logistic models, with uncertainty summarized using 95% credible intervals (CrIs).

### 2.4. Phase III. Robustness and Interpretation (Domains 8–10)

#### 2.4.1. Domain 8: Missing Data Handling

Run-time auditing procedures for robustness and interpretation (Domains 8–10)—Missing data rules were implemented as prespecified: insurance missing in 25% of encounters was retained as an unknown analytic category; sex missingness triggered complete-case enforcement across all models to support valid nested likelihood ratio tests. Temporal and stratified checks were conducted overall and by drug type across calendar years. Scope boundaries were enforced throughout: interaction analyses were prespecified as outside scope, and the workflow implemented the prespecified analytic plan only.

Missing data identification, imputation strategies considered, and analytic treatment of remaining missingness are described in Phase I, where they arise naturally within the workflow. The downstream impacts of these missing fields, particularly the non-random missingness of pain severity metrics and the health-system-level clustering of incomplete documentation, are contextualized within Section 4 to ensure complete analytic transparency.

Outputs were required to meet all prespecified verification criteria prior to interpretation (Table 1). These criteria were critical given the data quality limitations documented in Domains 3 and 4, including fragmented medication capture and high missingness in dose elements.

When execution errors occurred, error-informed prompt refinement was performed by rerunning revised code until all verification criteria were met, illustrating the iterative, bias-aware auditing process the framework supports. As noted above, a refinement was triggered when 308 records with missing sex were inadvertently included in models M1–M2, which initially prevented valid nested LRT comparisons. The code was updated to rigidly enforce a common complete-case sample across all models. Because overall missingness for this variable was less than 1%, imputation and additional sensitivity analyses were not warranted.

#### 2.4.2. Domain 9: Temporal and Stratified Checks

Sensitivity analysis and stratification—PRN opioid orders were segregated and evaluated separately from the primary analytic sample due to their distinct clinical indication within the clinical workflow and substantial volume, accounting for 41.3% of 209,889 valid opioid prescription orders (Figure 3a). Stratified analyses (using admission year) were executed within the analytic pipeline to monitor temporal consistency in PRN ordering patterns across the longitudinal study window (2014–2021). Because the Cerner RWD lacks a direct relational link to confirm whether an PRN order resulted in an administered dose, this operational workflow was treated as an unresolvable data-source constraint on the interpretation of ordering versus true administration patterns.

Temporal ICC estimation—To evaluate temporal variance patterns in overall PRN ordering, the framework supported calendar year-stratified, intercept-only logistic GLMMs. ICCs were calculated on the latent logistic scale (ICC_k_ = σ^2^_k_/σ^2^_total_), where σ^2^_total_ represents the sum of all hierarchical variance components plus the fixed logistic-distribution variance (π^2^/3 = 3.29). Because year-stratified sub-models are highly vulnerable to sparse data anomalies and boundary behavior, year-specific ICCs with 95% confidence intervals were generated using parametric bootstrapping rather than standard frequentist approximations.

#### 2.4.3. Domain 10: Scope Boundaries

Prespecified analytic claims—Inferential boundaries were hard-coded into the framework prior to analysis to prevent *post-hoc* overreach. Interaction analyses were prespecified as “out-of-scope” and are reserved for future work. Case-mix and system-level covariates were incorporated to assess crude vs. adjusted ORs; however, covariate-specific adjusted ORs were not reported because inference regarding these individual effects was outside the scope of the present study.

Study size and precision—Because this stage of the framework application was descriptive rather than inferential, the sample size was not derived from an *a priori* power calculation. Consequently, effect-size estimation and precision were constrained to broad contextual effects within clusters. The framework flagged that statistical power may be limited for sparse subgroup comparisons and highly parameterized models. However, future outcome-focused testing can pre-specify objective-specific sample sizes and power calculations, particularly for low-frequency demographic or clinical subgroups.

In-scope results—All results are strictly bound to the primary analytic sample of 16,734 pediatric cancer patients (76,395 encounters), excluding PRN orders, and are limited to the pre-specified estimands defined in Domain 1.

Modeling effects—Prespecified models for hospitalization opioid ordering (Y/N) were estimated overall and by drug type across calendar years, with adjusted effects, variance components, and ICCs summarized in Supplemental Table S4. Likelihood ratio testing supported meaningful improvement in model performance with covariate adjustment: compared with the random-effects-only baseline (M1), adding system-level factors (M2) only slightly improved fit (AIC: 92,347 → 92,344), while incorporating patient case-mix substantially improved predictive validity (AIC: 89,680), with the fully adjusted model yielding the lowest AIC and significant improvement over baseline (AIC: 89,673; LR test vs. M1, *p* < 0.001).

Residual variability—In the fully adjusted model, most residual variability in opioid ordering reflected differences among patients within the same health system (ICC = 19.9%), while between-health system variability was comparatively small (2.0%) after accounting for measured system-level and case-mix covariates. The encounter-within-patient variance component was negligible (<0.001%), consistent with guidance that variance components estimated at or near the boundary of zero do not substantially contribute to model fit and may be removed for parsimony [28,29]; therefore, the encounter-level random effect was omitted, resulting in a two-level model that retained the substantive sources of clustering while avoiding an unnecessary variance component. Although generated code could identify or encounter analytic failures such as model singularity, it did not necessarily determine the most appropriate subsequent analytic approach without expert methodological input. This result is important because it highlights the potential opportunity for automated expert statistical input in auditing.

### 2.5. Bias Assessment

Potential biases in defining opioid exposure and estimating prescribing patterns using the Cerner RWD were systematically identified and addressed across the eight primary bias categories depicted in Figure 4: selection, measurement, misclassification, systematic, temporal, missing data, confounding, and geospatial. The core contribution of this framework is to integrate these established, foundational bias-assessment principles [30] directly into the AI-assisted analytic workflow, linking them to explicit verification, stop/flag conditions, and run-time expert-review steps. Key empirical findings from the bias assessment are described below, and mitigation strategies for each category are visually summarized in Figure 4.

Selection bias—Selection bias was addressed as a foundational concern across Domains 1–4 of the analytic framework, because cohort construction and opioid order extraction involved eligibility-based exclusions that may not have been uniformly distributed across the hierarchical data structure. For the encounter-level binary exposure estimand, exclusion of hospitalizations exceeding 30 days and restriction to non-PRN orders may disproportionately affect higher-complexity patients and those receiving as-needed pain regimens, respectively, with potential implications for both adjusted effect estimates and variance component estimands. Restriction to the five most prevalent opioid types, which accounted for 96.9% of opioid orders, may also differentially affect exposure ascertainment across drug classes.

Representativeness was evaluated through exclusion audits stratified by cluster level, drug type, and calendar year, including checks of variation in PRN prescription exclusions across health systems and over time. Uniform application of eligibility criteria across health systems was verified to support the stability and interpretability of between-system ICC estimates. Future framework iterations will expand this workflow to compare patients retained versus excluded from the final analytic cohort across health system factors and patient case-mix characteristics, including age, sex, race/ethnicity, payor, admission diagnosis, admission type, and length of stay.

Misclassification bias—The initial medication extraction captured orders for 13 opioid types, from which five primary opioids with a sole active ingredient—morphine, fentanyl, oxycodone, hydromorphone, and methadone—were retained for exposure assessment. The remaining eight opioids lacked direct clinical indication for managing acute cancer-related pain and were excluded [31], as were combination products containing one of the five primary opioids alongside another active ingredient (e.g., fentanyl-bupivacaine). Potential under-capture from omitted trade names was evaluated via *post-hoc* random sampling of 10,000 prescription orders from a broader pediatric RWD cohort across the concurrent eight-year period. Overall, 6.7% of sampled orders were excluded due to missed trade name identification (Supplemental Table S3) [32,33,34]. Misclassification was concentrated in the hydromorphone category, where 23.2% of sampled prescriptions were listed under the trade name Dilaudid, although this estimate may be inflated given it was derived from a broader patient sample. Misclassification was negligible for morphine (<0.1%) and minimal for fentanyl (1.3%) and oxycodone (2.2%). Expanding extraction criteria to include trade names within the specific study cohort was not scoped in the original study proposal but is recommended for future analyses.

Measurement bias—To reduce potential overestimation of opioid exposure, PRN orders were excluded from primary analyses, with the appropriateness of this decision evaluated empirically through discontinuation rate comparisons. The magnitude of the impact of this exclusion on the study objective was assessed via differences in discontinuation status between PRN and non-PRN orders to identify and mitigate masking bias in the assessment of opioid exposure at the encounter level. Among 86,636 PRN orders, 87.7% were discontinued, compared to 15.5% of the 123,255 non-PRN orders comprising the primary analytic sample. After adjustment for drug type, as-needed orders had 4.34 times higher odds of discontinuation (95% CI: 4.31, 4.37, *p* < 0.001), consistent with a distinct clinical documentation process governing PRN orders within the EHR, either recorded separately upon transitioning to completed status or documented infrequently relative to actual administration. This differential discontinuation pattern supports exclusion of PRN orders from primary analyses, promoting homogeneity in the assessment of opioid exposure at the encounter level.

Systematic bias—Variation in data-entry practices across health systems was addressed through hierarchical modeling of clustering at the health system, patient, and encounter levels, with ICCs estimated at each level as described in Domain 5.

Temporal bias and health system drift—PRN opioid orders were relatively stable across the study period, with the highest rate observed for oxycodone (69.2%; Figure 3a). However, between-system variation in PRN order patterns declined substantially over time, with the ICC decreasing from 20.2% (95% CI: 10.5%, 29.5%) in 2014 to 10.0% (95% CI: 5.1%, 14.6%) by 2021 (Figure 3b). This downward trend likely reflects advances in EHR documentation, data entry standardization, and stricter opioid prescription monitoring. By contrast, patient- and encounter-level ICCs remained relatively stable throughout the study period (range: 5.5–9.3% and 3.3–7.1%, respectively), suggesting that early health system-level habits in PRN ordering no longer dominated the decision. Instead, the decisions appeared to be more tailored to the induvial patient and their clinical condition at a point in time.

Missing data bias—Complete dosage information was available in only 35% of opioid orders and was formally assessed across years and cluster levels, as described in Domain 4. Insurance status, missing in 25% of encounters, was retained as an unknown analytic category in outcome models. Due to inconsistent measurement and approximately 25% encounter-level missingness, pain was limited to descriptive analyses. Missingness across other case-mix variables was less than 5%.

Confounding bias—Opioid exposure parameterization and valid-order identification were evaluated across cluster levels, drug types, and calendar years. Outcome models were specified with adjustment for health system factors and patient case-mix as described in Domain 5.

Geospatial bias—Although geospatial bias is sometimes treated as a dimension of confounding, it was addressed separately in this workflow given the recognized limitations of geographic data in the Cerner RWD and implications for social determinants of health (SDOH). Geospatial bias assessment was constrained by the limited spatial granularity available, where only the first digit of the patient’s ZIP code was accessible, precluding neighborhood-level evaluation of SDOH. Geospatial variation was addressed indirectly through hierarchical modeling, with geographic region fixed effects spanning nine U.S. regions and health system random effects serving as proxies for unmeasured spatial variation in opioid ordering patterns.

### 2.6. Portability Across LLMs: Key Domain Findings and Adaptations

To assess workflow portability, the structured analytic specifications were independently evaluated using Claude (Anthropic) alongside the baseline ChatGPT-5.2 (OpenAI) workflow. No clinical data were shared with either LLM; generated R code (v 4.4.2) was executed locally. The cross-LLM assessment revealed that reproducibility depends on the rigor of upstream specifications, but it ultimately yielded concordant statistical results once estimation settings were harmonized.

Specification and data inputs (Domains 1–4). Across the domains Objective and estimands, Data inputs and outputs, Cohort and filters, and Variable coding, portability depended strongly on the precision of the upstream analytic specification. Syntactical ambiguity required increased prompting to clarify analytic intent. Reproducible code generation required explicit, priori definitions of the following: (1) measurement levels, category references, and exact string configurations; and (2) missing-data rules, clustering identifiers, and the exact unit of analysis.

Model specification and data inputs (Domains 5–7). The LLMs introduced safeguards at different stages of the analytic pipeline. For estimation settings, ChatGPT utilized the precise Laplace approximation (nAGQ = 1). Claude prioritized computational speed via the nAGQ = 0 approximation. Despite distinct default approximations, the resulting overall model ICCs were highly stable. Patient-level ICCs matched at 19.9% vs. 19.7%, and health-system-level ICCs matched at 2.0% vs. 2.2% (an absolute difference of 0.002). Claude implemented automated upstream screening (flagging fewer than 5 events per health-system stratum or fewer than 10 events overall) to catch sparse outcomes like Methadone and Hydromorphone before model execution. ChatGPT relied on downstream diagnostics (isSingular (…, tol = 1 × 10^−5^) vs. Claude’s tol = 1 × 10^−4^) and post-hoc expert review.

Robustness, missing data, and estimation (Domains 8–10). Both models successfully preserved the prespecified missing-data rules. Both independently enforced complete-case logic on missing sex data (<0.5% missingness) to maintain valid nested likelihood ratio tests. When bootstrap parameters were unmeasured, ChatGPT default-initialized 1000 replications (higher stability, high computing cost), whereas Claude selected 200 replications for parsimony.

Final verification and equivalence—Once estimation criteria and bootstrap parameters were explicitly standardized across both platforms, the reduced M4 models generated via ChatGPT and Claude produced identical random-effect variance estimates, ICCs, fixed-effects, and AIC values evaluated through the third decimal place; fixed-effect estimates similarly matched across adjustment factors but are reserved for a separate clinical manuscript. Cross-LLM concordance confirms framework portability while proving that successful code execution alone does not replace expert statistical review for model interpretation. The cross-LLM portability exercise demonstrates that analytic reproducibility relies on structured process safeguards rather than identical code generation or automatic estimate equivalence. In summary, standardizing specifications across Claude and ChatGPT produced concordant M4 random-effect variance estimates, ICCs, and AIC values, while revealing implementation differences in bootstrap replications and sparse-data screening.

## 3. Discussion

Study contributions and empirical value—This study demonstrates a 10-domain analytic framework with an embedded, run-time AI semantic auditing loop, addressing a critical gap in computational modeling of RWD: the need to shift from passive *post-hoc* disclosure to active run-time verification. Current pre-processing templates and checklists form the backbone of this effort, but in lieu of reporting data relevance and quality retrospectively, our approach operationalizes these reporting expectations directly into an executable, active run-time gatekeeper. Tested on a feasibility cohort of 16,734 pediatric cancer patients (76,395 encounters) across 55 U.S. health systems in the Cerner RWD, the framework maps prespecified analytic goals directly to a closed-loop code auditor to check for logic flaws, bias, and vulnerabilities during execution.

Systematic application of the framework’s phased architecture revealed critical data features and structural boundaries prior to final modeling. In Phase I, the framework exposed data limitations early, demonstrating that total daily dose could not be validly estimated from the Cerner RWD. It established precise exposure definitions, showing five drugs captured 96.9% of all orders, and empirically justified separate tracks for PRN orders (which comprised 41.3% of orders and exhibited a 4.34-fold higher odds of discontinuation than non-PRN orders). In Phase II, the fully adjusted model successfully partitioned variance, revealing that patient-level variation (ICC = 19.9%) substantially exceeded institutional variation (ICC = 2.0%). Accounting for clustering at both the patient and health-system levels reduced the risk of attributing structural influences to individual-level factors and was consistent with findings previously reported in pediatric oncology [35,36]. In Phase III, longitudinal screening revealed divergent opioid-ordering trajectories from 2017 onward, with decreasing morphine and increasing fentanyl ordering (Table 3A,B). It also tracked a drop in health-system clustering for PRN orders from 20.2% in 2014 to 10.0% in 2021, mapping real-world shifts in EHR standardization and clinical policy.

Our proposed framework builds on retrospective reporting checklists and pre-study planning approaches, including RECORD [12,14], STaRT-RWE [37], HARPER [38], and the framework described by Jaksa et al. [13], while extending these principles toward a comprehensive operational execution loop designed to support increasing automation. As summarized in Table 4, these approaches share important elements with our framework, including transparent cohort definition, estimand specification, data-quality assessment, and prespecified sensitivity analyses; however, our framework reorganizes and operationalizes these elements across 10 functional domains within an iterative analytic workflow. Rather than functioning solely as a reporting or planning aid, the framework serves as a run-time gatekeeper that maps prespecified analytic goals to a closed-loop LLM-based semantic auditing process capable of evaluating code logic, potential bias, and analytic vulnerabilities during execution. Within this architecture, the LLM functions as the semantic auditing engine rather than as an autonomous decision-maker, while expert statistical review remains responsible for methodological judgments that cannot be safely resolved through automated rules alone. In this way, the framework is intended to bridge clinical meaning and mathematical validity by requiring both to be evaluated within the analytic workflow rather than only after results are produced.

Run-time AI auditing and computational scalability—Acting as an active run-time gatekeeper, the curated AI-audited pipeline successfully enforced data quality rules during the code-drafting process. The semantic auditor caught critical execution flaws, such as the inclusion of 308 records with missing sex, automatically correcting the script to enforce a common complete-case sample required for valid nested likelihood ratio testing. Furthermore, once estimation criteria and bootstrap parameters were explicitly standardized, independent workflows across ChatGPT-5.2 and Claude Sonnet 5 achieved strict cross-LLM concordance, producing random-effect variance estimates, ICCs, and AIC values matching to the third decimal place.

To scale these computationally intensive generalized linear mixed models (GLMMs) over large hierarchical datasets, the framework operationalizes a tiered execution strategy. Investigators can leverage Generalized Estimating Equations (GEEs) to establish efficient population-averaged baselines and screen fixed effects upstream. This optimized foundation accelerates downstream GLMM variance partitioning, while packages like glmmTMB can be deployed for run-time sensitivity checks of complex random-effects structures. By transforming passive transparency checklists into an executable, bias-aware code auditing process, this framework establishes a scalable pathway toward full-stack automated RWD validation.

## 4. Limitations and Future Iterations

Several limitations in this study merit discussion and can be classified broadly as follows: (1) inherent constraints of the real-world data source that the framework could identify but not resolve; and (2) framework-application limitations involving analytical trade-offs during cohort selection that were identified through run-time evaluation but ultimately required expert judgment.

Data source constraints—First, regarding the electronic health record data source, using the Cerner RWD “drug” variable to identify active ingredients can underrepresent medication orders unless a comprehensive lexicon matches generic and trade names. Our exploration in a random sample of 10,000 pediatric patients suggested this issue is minimal (<0.3% of orders), with the notable exception of hydromorphone, where 23% of orders were hidden under the trade name Dilaudid (see Supplemental Table S3).

Second, Cerner’s tracking of PRN orders (41.3% of the database) introduced structural ambiguity, specifically when an PRN order was subsequently completed: the database logged the administration as a separate, unrelated data row that stripped the “As-needed” flag, breaking the relational link between the order and administration. We chose to exclude PRN orders based on clinical verified chart audits that completed orders do not contain the “As-Needed” flag. It should be noted PRN order percentages have not been reported in other studies [35,39]. The reliance on manual verification highlights that large database undertakings require continuous clinical guidance and inferential goals may be difficult to achieve [40].

Third, the Cerner RWD also severely limits geographic resolution to nine broad U.S. regions, obscuring state-by-state variations driven by local payor structures and preventing the integration of neighborhood-level metrics like the Child Opportunity Index (COI) [41]. While Davis et al. (2024) proposed extending the Cerner RWD to 3-digit ZIP codes via a Standardized Health Data and Research Exchange [42], our model was restricted to macro-regions, limiting assessment of more granular geographic and community-level contextual factors. In addition, potentially influential health-system and encounter-level characteristics, including staffing patterns, patient pain severity, and co-morbidity burden, were not consistently available and therefore remained unmeasured. These data-level gaps represent structural barriers that a governance framework can flag for transparency but may not be fully resolved through analytic and computational methods alone.

Framework application/cohort selection tradeoffs—The current application of our framework was limited by its reliance on analyst-defined, strict sample cut-off points for computational stability, specifically the following: (1) truncating each patient’s history at the 20th hospitalization; and (2) excluding hospital stays >30 days. These rules may disproportionately exclude high-utilization or prolonged-stay patients whom the model may intend to characterize, thereby introducing selection bias. Application of the framework made these cohort-definition decisions explicit and auditable, but the present audit did not independently determine optimal thresholds, formally compare alternative cut-points or apply prespecified rules for identifying when sparsity threatened hierarchical estimation. Future iterations could extend the cohort-definition audit by automatically characterizing encounter and LOS distributions, quantifying patient and tenant attrition across candidate thresholds, and assessing resulting model estimability and stability. Prespecified expert-consensus rules could then trigger sensitivity analyses and support ranking of alternative specifications according to their expected ability to mitigate selection bias while preserving estimability. Within a multi-agent LLM architecture, these diagnostics could inform, rather than replace, the final human-in-the-loop decision.

Automated statistical governance—The LLM in this case study was limited to reviewing targeted aspects of the analytic code and workflow that directly mapped to the framework domains, including model diagnostics, stop/flag conditions, and potential sources of bias requiring further verification or revision. However, model execution, final diagnostic assessment, and clinical interpretation remained strictly under analyst and biostatistical oversight. Future clinically focused iterations of this framework could deploy an AI council in numerous ways, e.g., by characterizing patterns of pain-score missingness across health systems, clinical settings, opioid exposure, and patient characteristics. Then, we could deploy automated evaluation of which missing-data approaches are supportable given the observed missingness mechanism. Similarly, automated sensitivity analyses could consider alternative pain-summary measures that account for differences in assessment frequency and timing, rather than relying solely on the maximum recorded score. These evaluations were beyond the scope of the current case study but represent future methodological steps that could be incorporated. We also recognize that formal methods for weighing the magnitude and direction of competing biases within AI-assisted workflows represent a key area for future methodological development that warrants dedicated investigation.

No patient-level or identifiable data were shared with the LLM; all use of AI-assisted code generation was conducted in accordance with the approved IRB protocol and institutional data-governance requirements. Future applications should expand the AI’s role. Utilizing institutionally contained AI systems, restricted-access computing environments, and deidentified or synthetic/cloned datasets will allow collaborative LLM expert councils to safely run automated sensitivity analyses, optimize cohort filters and mathematically weigh competing biases without exposing protected clinical data. We view these automated approaches as complementary to, rather than substitutes for, institutional privacy, security, and regulatory requirements.

## 5. Conclusions

The 10-domain framework provides a structured, reproducible mechanism for governing RWD analyses by embedding a closed-loop semantic code auditor and run-time analytics directly into the data science workflow. By linking prespecified analytic goals to transparent exposure definition, systematic bias assessment, explicit verification, and expert-review triggers, the framework moves beyond passive transparency checklists toward an executable, bias-aware code-auditing process.

Its application to the Cerner RWD demonstrated the practical value of this approach. The framework identified consequential data constraints before final analysis, including the inability to derive reliable daily medication doses or morphine milligram equivalents from order-level data alone, while also flagging unmeasured confounding and analytic decisions requiring expert judgment. Cross-LLM concordance in identifying problematic logic and reproducing complex workflows further supports the portability of this run-time gatekeeper.

However, because this initial application still relied on analyst-defined sample thresholds and other expert-informed trade-offs to maintain computational and statistical stability, future iterations should incorporate more automated statistical diagnostics and prespecified decision-support rules while retaining clinical and statistical oversight. Multi-agent LLM architectures could support an expert statistical council by performing sensitivity analyses of missing-data mechanisms, evaluating statistical approaches for heavy-tailed healthcare utilization data, and systematically weighing competing sources of bias. Within institutionally governed AI systems, secure computing environments, and IRB-compliant synthetic-data workflows, these functions could be implemented without exposing protected clinical information. Coupling automated verification with structured human oversight could strengthen the reproducibility and scientific rigor of observational research and, over time, inform publication standards that encourage framework-driven, audited validation before peer review. Together, these advances offer a scalable path toward more trustworthy, transparent, and clinically meaningful RWE generation as AI becomes increasingly embedded in analytic workflows.

## Figures and Tables

**Figure 1:**
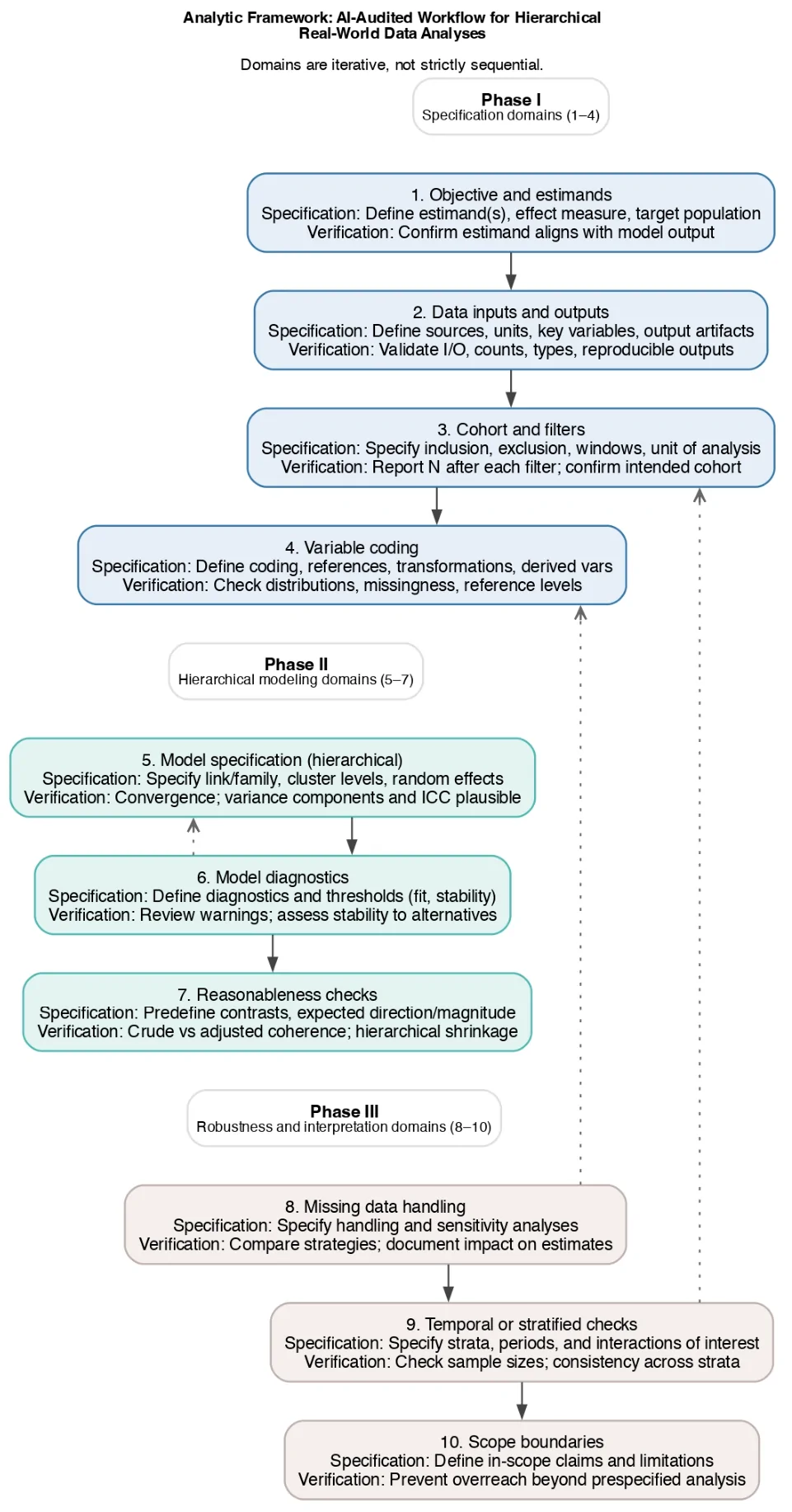
Analytic framework for AI-assisted first iteration code generation in hierarchical RWD analyses. Colors differentiate the three phases, and arrows indicate the general workflow sequence across 10 domains, which are iterative, rather than strictly sequential.

**Figure 2:**
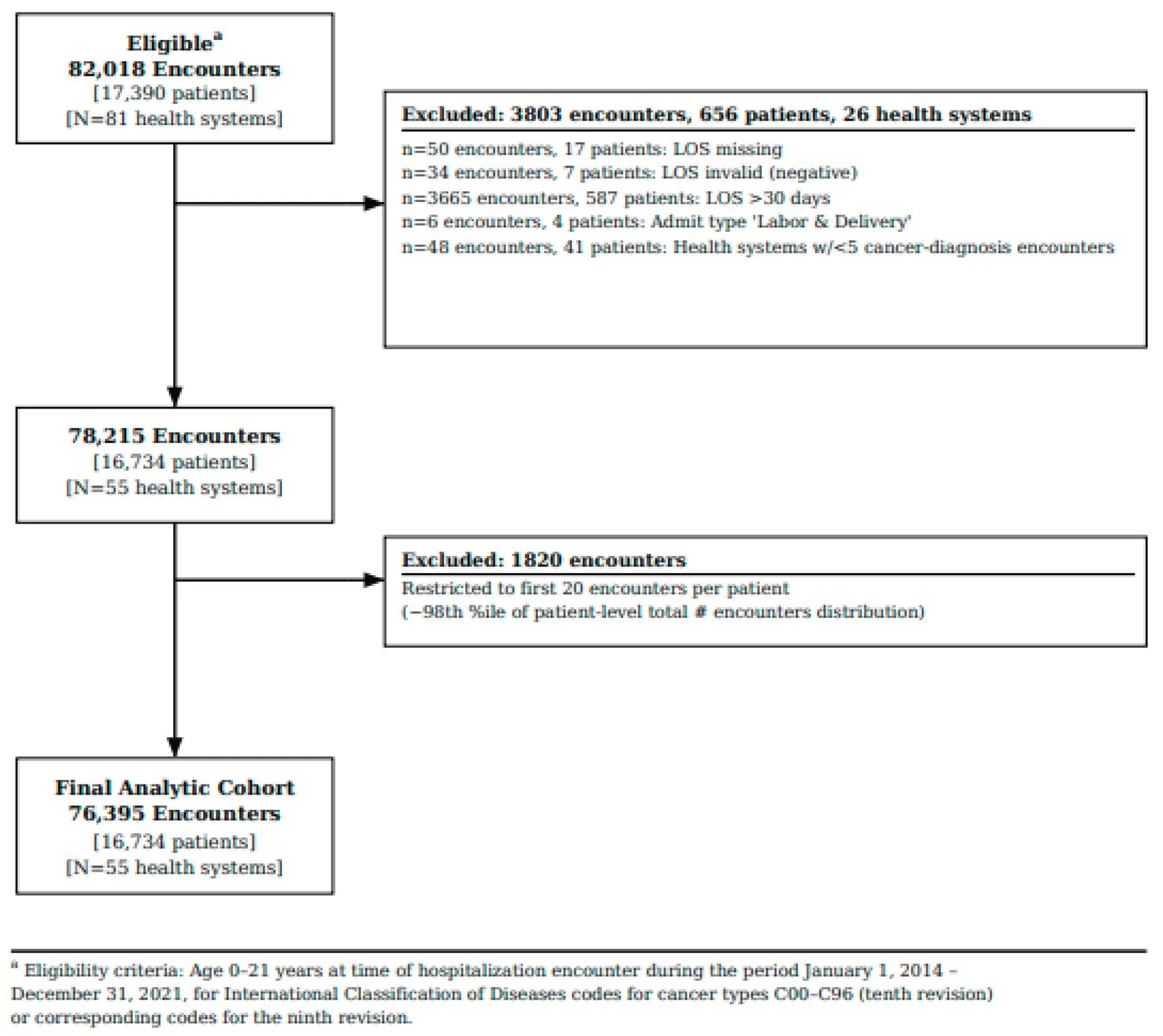
Flow diagram displaying selection of encounters, patients, and health care systems for analyses.

**Figure 3:**
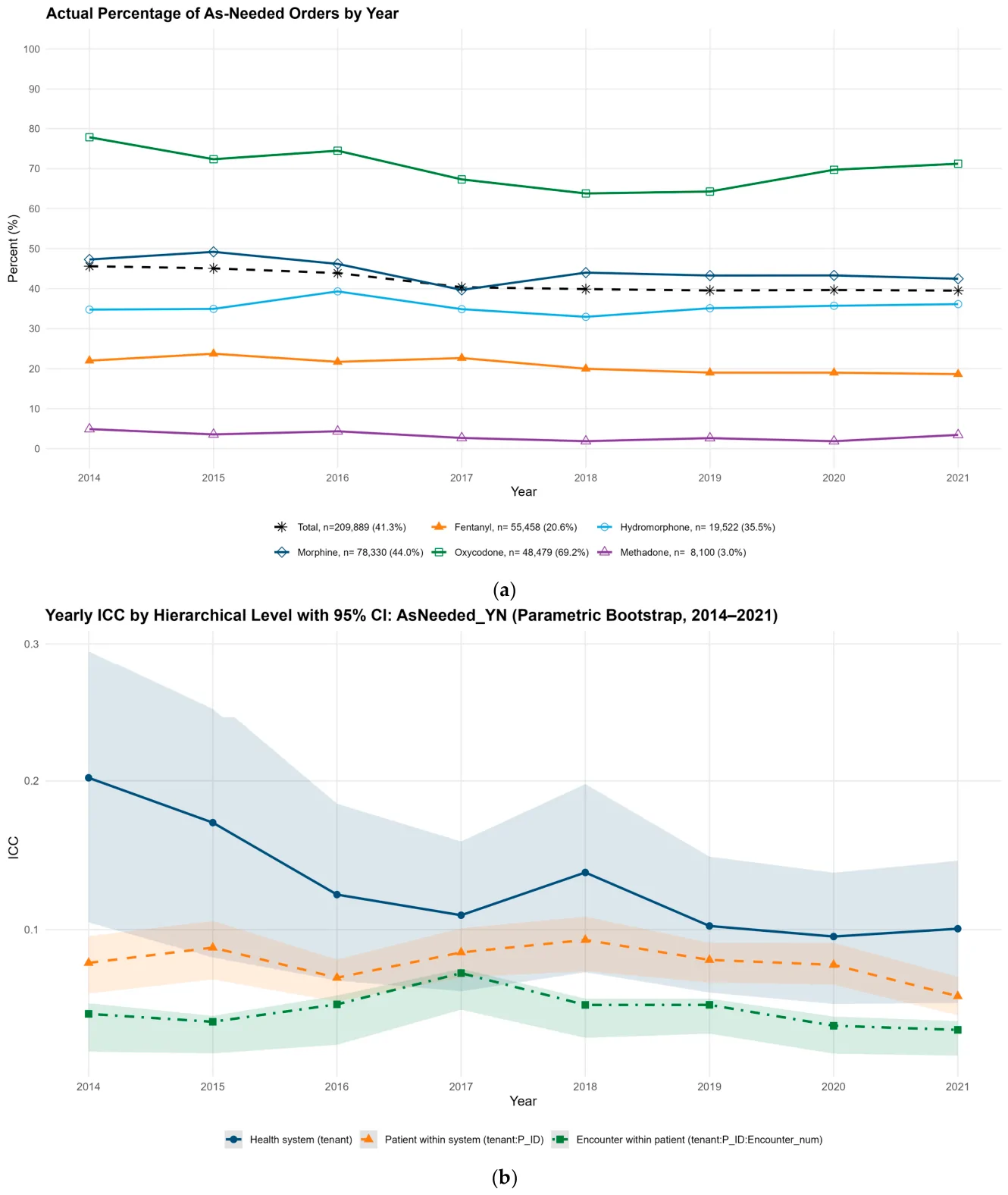
(**a**) Descriptive comparison of opioid order characteristics by as-needed (PRN) status (included vs. excluded orders) overall and by drug type. (**b**) Hierarchical variance decomposition for PRN status (excluded vs. non-PRN) using multilevel logistic models: variance components and ICCs by level and year. Note. Variance components (σ^2^) and intraclass correlation coefficients (ICCs) were estimated from multilevel logistic models with random intercepts for health systems, patients nested within health systems, and encounters nested within patients. The ICC represents the proportion of total variance attributable to each grouping level, with the logistic residual variance treated as fixed at π^2^/3 (=3.29). Models included a fixed effect for opioid drug type; year-stratified models were fit to evaluate temporal patterns in ICC estimates. One health system with only two opioid orders (no outcome variation) was excluded from model-based estimation.

**Figure 4:**
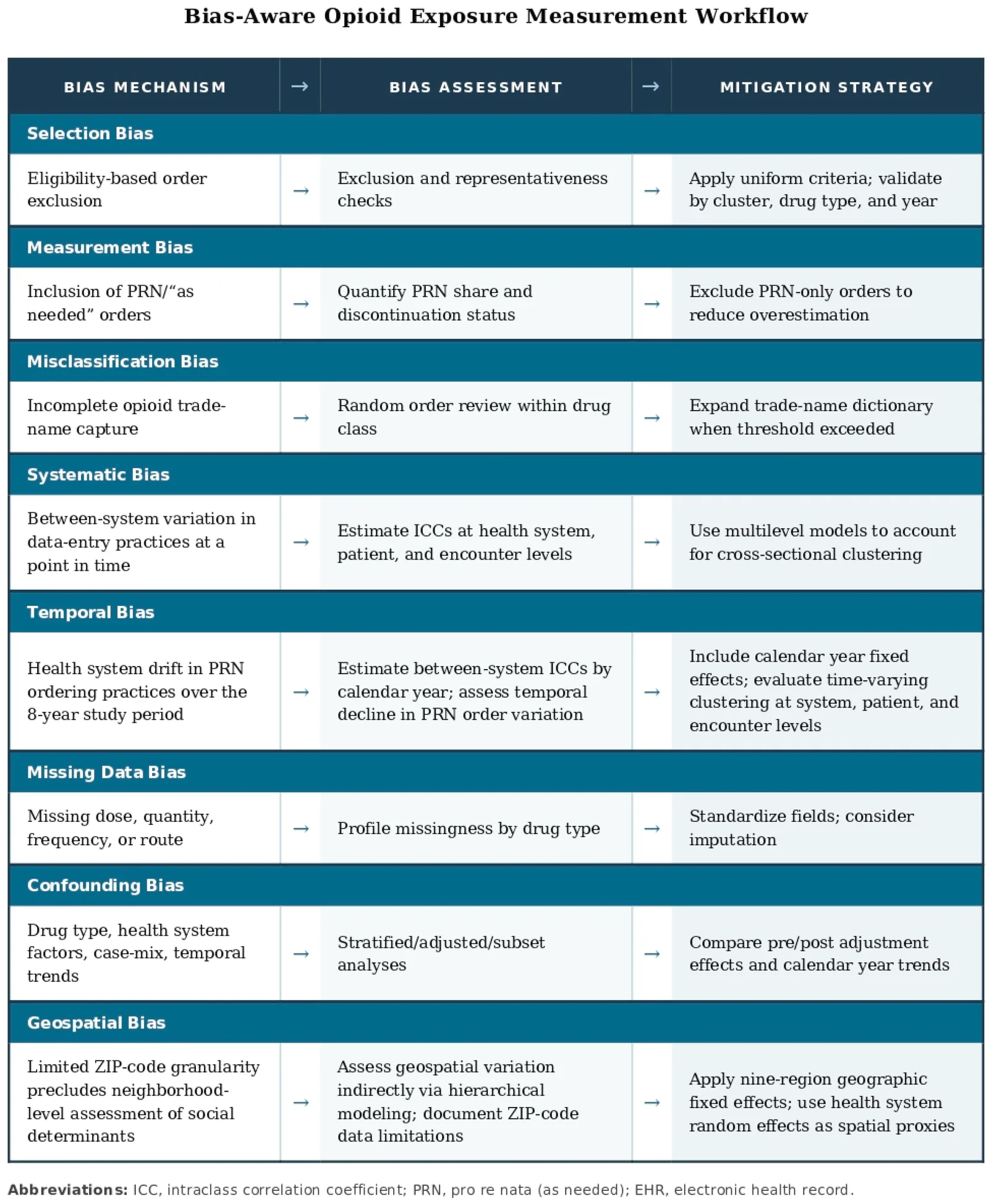
Systematic identification and address of potential biases in defining and examining opioid exposure using the Cerner RWD. Arrows denote the sequential workflow from bias identification through assessment to mitigation.

**Table 1:** Structured prompt framework for AI-assisted first-iteration code generation in hierarchical real-world data analyses applied to exemplar (opioid order during hospital encounter, Y/N).

#	Domain	Prompt Template Element	Required Specification	Verification Before Interpretation
Phase I. Specification (Domains 1–4)				
**1**	**Objective and estimands**	“Write [language] script to estimate [outcome] with [effect estimands] and [variance estimands].”	Co-primary estimands specified a priori: (1) aORs for encounter-level outcome across prespecified covariates; (2) cluster-level variance components and ICCs for each level of the prespecified hierarchy (e.g., health system, patient, encounter).	Output includes aORs with 95% CIs and *p*-values, model fit statistics (AIC), variance components, and ICCs for each prespecified model (M1–M4).
**2**	**Data inputs and outputs**	“Read [input path], write outputs to [directory/prefix], export [tables/figures].”	Exact local data import path; output destination directory; file prefix (e.g., R_Output_); traceable export naming conventions; required output formats (tables, figures).	All prespecified output files generated with filenames traceable to the analysis run; I/O counts and variable types validated against the data dictionary.
**3**	**Cohort and filters**	“Apply [inclusion/exclusion criteria] in this order and print N after each step.”	Ordered inclusion/exclusion criteria; unit of analysis (e.g., encounter-level); prespecified truncation rules (e.g., >20 encounters per cluster) and minimum system-level count rules; expected sample-size counts (N) for validation.	Analytic N reconciles with cohort flow at each sequential filter step; printed N at each step confirms intended cohort before modeling.
**4**	**Variable coding**	“Use exact variable names/types; set [reference levels]; run distributional checks across covariates and outcome.”	Case-sensitive variable names; data types (continuous vs. factor); binary coding rules; factor reference categories; derived variable definitions; outcome specification (Y/N binary).	**Initial verification:** Levels, references, and recodes matched the data dictionary and specification sheet; distributions were checked before modeling. **Iterative safeguard:** Retrospective review identified low-cost event/non-event checks across outcome, health system, calendar year, and health system × year strata as candidates for prospective pre-fit automation to flag sparse or no-variation cells before model fitting.
**Phase II. Hierarchical Modeling (Domains 5–7)**				
**5**	**Model specification**	“Fit prespecified model sequence M1–M4 with [hierarchy/covariance structure/covariates].”	Logistic GLMMs with nested hierarchy; four prespecified models: M1: random effects only (health system, patient, encounter); M2: M1 + system-level factors; M3: M2 + patient case-mix; M4: M3 + calendar year (fully adjusted).	Formulas and covariate blocks match the prespecified analysis plan; LR tests comparing M2–M4 to M1 use a common complete-case sample across all models. ^a^
**6**	**Model diagnostics**	“Return convergence, singularity, fit statistics, ICCs, variance components, runtime, and explicit stop/flag criteria; re-run diagnostics after any model modification.”	Convergence and singularity checks; AIC/model comparison; variance-component and ICC extraction per cluster level; runtime logging; residual/influence diagnostics where applicable; explicit stop/flag criteria for boundary variance, instability, or nonconvergence; re-verification after expert-guided model modification.	**Automated flag:** Full M4 was singular, with encounter-level variance essentially zero (8.85 × 10^−13^). **Expert-guided iteration:** The encounter level was removed after the review of the hierarchy. **Re-verification:** The reduced model was nonsingular; patient- and health-system variance components were unchanged, fixed-effect estimates were essentially identical (maximum absolute OR change: 0.027%), and fixed-effect-only predicted probabilities were nearly identical (r = 0.99999999). The reduced pathway was then retained for subsequent analyses.
**7**	**Reasonableness checks**	“Provide crude and adjusted summaries for key contrasts; report cluster-level summaries by calendar year; quantify event/non-event support overall and across hierarchical/time strata before computationally intensive fitting.”	Prespecified crude rates/effect estimates and cluster-year summaries, supplemented after first-pass review by automated counts of events/non-events overall, by health system, by year, and by health system × year; flag zero-event, no-variation, and low-information strata for expert review rather than automatically selecting an alternative estimator.	**Retrospective automation check:** Methadone showed pronounced sparsity (1.95% events; 52.7% of health systems and 63.9% of health-system-years had zero events; 80.8% of health-system-years had <5 events). Hydromorphone also had substantial low-information health-system-year cells (58.5% with <5 events). **Interpretive guardrail:** Pre-fit distributions can flag elevated estimation risk and move repetitive verification upstream, but did not by themselves determine the appropriate estimator; methodological response remained expert-led.
**Phase III. Robustness and Interpretation (Domains 8–10)**				
**8**	**Missing data handling**	“Implement [variable-specific missing-data rules]; flag records excluded from LR tests due to missingness; run [sensitivity analyses] if prespecified.”	Variable-specific strategy defined a priori (e.g., “unknown” insurance coded as analytic category; sex missing → complete-case for all models to enable valid nested LR tests). Sensitivity analyses run when prespecified; missingness threshold for imputation defined a priori (e.g., >5%).	Missing-data rules applied consistently; complete-case sample used uniformly across M1–M4 to support valid LR test comparisons; proportion missing documented; imputation performed only if prespecified threshold exceeded; deviations from plan recorded with rationale.
**9**	**Temporal and stratified checks**	“Run fully adjusted model stratified by [strata variable]; export [plots/tables]. If pre-fit or model diagnostics are flagged, return the flag and candidate analytic options for expert review; encode the approved alternative and re-verify.”	Fully adjusted models were estimated overall and by drug type across calendar years. The initial frequentist GLMM pathway retained the prespecified hierarchy. First-pass diagnostics and subsequent expert review informed two reusable modifications: (1) removal of the encounter-level random intercept when its estimated variance approached the boundary, to reduce model complexity without important information loss; and (2) use of Bayesian multilevel estimation with weakly informative priors for methadone and hydromorphone after sparse-data and model-instability concerns were identified during iterative review.	**Flag and expert decision:** Instability in the methadone and hydromorphone models prompted expert methodological review; Bayesian multilevel estimation with weakly informative priors was selected and then encoded for consistent reuse. **Re-verification:** Posterior sampling checks showed no divergent transitions or maximum tree-depth warnings; the maximum observed tree depth was 6 in both final Bayesian fits. **Efficiency value:** Automating inexpensive upstream checks can identify models at elevated risk for instability before computationally intensive estimation, reducing potentially unnecessary or prolonged model-fitting attempts while retaining expert oversight of alternative analytic strategies.
**10**	**Scope boundaries**	“Implement prespecified analyses only; flag any scope expansion before execution.”	Workflow implements the prespecified analytic plan as an exemplar of AI-assisted code generation and iterative auditing. AI is used to translate analyst specifications and expert-reviewed methodological decisions into reproducible code, automated checks, flags, and approved alternative pathways; unsanctioned model expansion is prohibited. All data processing, model fitting, and interpretation remain investigator-led in a secure local environment.	Outputs remained within prespecified scope; deviations and expert-approved modifications were documented. The exemplar demonstrates value beyond code drafting: once an analytic problem was adjudicated by the biostatistician, AI-assisted iteration converted that decision into reusable computational safeguards for subsequent models and future applications. PHI and clinical data were not shared with the AI system; code generation/refinement only.

aOR, adjusted odds ratio; AIC, Akaike information criterion; GLMM, generalized linear mixed model; ICC, intraclass correlation coefficient; LR, likelihood ratio. ^a^ Guardrail prompt: “Use the same estimation method and complete-case dataset across all compared models; flag any non-nested or otherwise non-comparable model comparisons before reporting LR test results.”

**Table 2:** Analytic inputs, operational definitions, and planned use.

Domain	Variable	Operational Definition or Parameterization	Analytic Use
**Patient characteristics**	Age	Categorized as 0–1, 2–12, 13–17, and 18–21 years.	Candidate predictor
	Sex	Male or female.	Candidate predictor
	Insurance type	Public, private, or other.	Candidate predictor
	Race and ethnicity	American Indian/Alaska Native, Asian/Asian American, Black/African American, Hispanic/Latino, White, mixed race, other, or unknown/preferred not to state.	Candidate predictor
**Clinical characteristics**	Cancer diagnosis	First recorded cancer diagnosis classified into eight clinically derived categories using ICD-10 codes C00–C96 or comparable ICD-9 codes.	Case-mix adjustment
	Admission type	Elective, emergency, routine, urgent, or other/unknown.	Candidate predictor
	Maximum inpatient pain score	Maximum daily recorded numeric score from 0–10; the assessment instrument could vary across institutions and clinical settings.	Measurement heterogeneity across sites and approximately 25% missingness
**Health-system characteristics**	Health-system volume	Low, moderate, or high based on interquartile range	System-level predictor
	Geographic region	Nine U.S. geographic regions.	System-level predictor
	Admission year	2014–2021; treated categorically or used in stratified analyses.	Temporal adjustment or stratification
**Opioid-order characteristics**	Opioid exposure	Any inpatient opioid order versus none; coded 1 = exposed and 0 = unexposed.	Primary outcome
**Data-processing specifications**	Eligibility and filtering	Prespecified inclusion, exclusion, and analytic-population criteria applied before descriptive analyses and modeling.	Cohort construction
	Missingness and distribution checks	Missingness, sparse cells, ranges, skewness, outliers, and possible data-entry concerns evaluated before modeling.	Data-quality assessment
	Continuous-variable assessment	Histograms, boxplots, and, when appropriate, predictor–outcome plots; linearity assessed for continuous specifications.	Functional-form assessment
	Output conventions	Prespecified output directory, consistent file prefix, and traceable names for checks, tables, figures, models, and fit statistics.	Reproducibility and audit trail

Abbreviations: ICD-9, International Classification of Diseases, Ninth Revision; ICD-10, International Classification of Diseases, Tenth Revision.

**Table 3:** (**A**) Overall only (yearly raw prevalence) and adjusted probability ^a^ of opioid ordering at the encounter by year and drug type. (**B**) Fully adjusted odds ratios for calendar-year effects on opioid ordering during encounters, overall and by drug-specific outcome ^b^.

(**A**)							
**Year**	**Observed Prevalence**	**Overall**	**Morphine**	**Fentanyl**	**Oxycodone**	**Hydromorphone**	**Methadone**
	n (%)	p̂ (95% CI)	p̂ (95% CI)	p̂ (95% CI)	p̂ (95% CI)	p̂ (95% CrI)	p̂ (95% CrI)
**Total**	76,087 (33.5%) ^c^						
2014	6760 (34.1%)	31.9% (28.5, 35.4)	13.8% (11.8, 16.1)	15.3% (12.6, 18.4)	2.3% (1.5, 3.5)	5.4% (0.8, 17.9)	2.2% (0.6, 7.7)
2015	8312 (33.4%)	32.4% (29.1, 35.9)	12.6% (10.7, 14.7)	15.9% (13.2, 19.1)	2.3% (1.5, 3.6)	4.8% (0.7, 15.8)	2.9% (0.9, 9.0)
2016	9936 (33.1%)	32.1% (28.9, 35.6)	13.0% (11.1, 15.1)	16.2% (13.4, 19.4)	2.1% (1.4, 3.2)	4.9% (0.7, 16.1)	1.7% (0.4, 6.4)
2017	10,130 (33.5%)	32.7% (29.4, 36.1)	12.4% (10.6, 14.5)	17.0% (14.2, 20.4)	2.5% (1.6, 3.8)	4.9% (0.7, 15.8)	1.8% (0.5, 6.6)
2018	10,669 (32.1%)	31.8% (28.6, 35.2)	11.3% (9.6, 13.2)	17.2% (14.3, 20.6)	2.3% (1.5, 3.6)	4.7% (0.6, 15.8)	1.3% (0.4, 4.9)
2019	10,855 (32.0%)	31.3% (28.1, 34.7)	11.4% (9.7, 13.4)	17.0% (14.2, 20.3)	2.4% (1.6, 3.7)	5.3% (0.8, 17.3)	1.7% (0.4, 6.2)
2020	9520 (34.7%)	34.5% (31.1, 38.0)	11.3% (9.6, 13.2)	19.4% (16.2, 23.0)	2.4% (1.5, 3.6)	5.9% (1.0, 18.6)	2.0% (0.5, 7.7)
2021	9905 (35.5%)	34.3% (30.9, 37.8)	11.8% (10.1, 13.8)	19.3% (16.1, 22.9)	2.3% (1.3, 3.2)	6.3% (0.9, 20.9)	2.2% (0.6, 9.0)
(**B**)							
**Year**	**Overall**		**Morphine**	**Fentanyl**	**Oxycodone**	**Hydromorphone**	**Methadone**
	aOR (95% CI)		aOR (95% CI)	aOR (95% CI)	aOR (95% CI)	aOR (95% CrI)	aOR (95% CrI)
2014	Ref		Ref	Ref	Ref	Ref	Ref
2015	1.02 (0.94, 1.12)		0.90 (0.81, 0.99)	1.05 (0.95, 1.16)	1.01 (0.86, 1.18)	0.95 (0.78, 1.14)	1.54 (1.05, 2.28) ^^^
2016	1.01 (0.93, 1.10)		0.93 (0.84, 1.03)	1.07 (0.97, 1.19)	0.92 (0.78, 1.08)	1.03 (0.83, 1.26)	0.96 (0.62, 1.50)
2017	1.04 (0.95, 1.13)		0.88 (0.80, 0.98) *	1.14 (1.03, 1.26) **	1.07 (0.92, 1.25)	1.01 (0.82, 1.23)	1.03 (0.66, 1.56)
2018	1.00 (0.91, 1.09)		0.79 (0.72, 0.88) **	1.16 (1.05, 1.27) **	1.03 (0.88, 1.20)	1.08 (0.88, 1.33)	0.65 (0.41, 1.03)
2019	0.97 (0.89, 1.06)		0.81 (0.73, 0.89) **	1.14 (1.03, 1.26) **	1.05 (0.90, 1.22)	1.22 (1.00, 1.48)	0.96 (0.61, 1.50)
2020	1.12 (1.03, 1.23) *		0.80 (0.71, 0.89) **	1.33 (1.21, 1.47) **	1.03 (0.88, 1.21)	1.32 (1.09, 1.61) ^^^	1.60 (1.01, 2.54) ^^^
2021	1.11 (1.02, 1.22) *		0.84 (0.75, 0.93) **	1.33 (1.20, 1.47) **	0.90 (0.77, 1.06)	1.61 (1.32, 1.95) ^^^	1.96 (1.22, 3.07) ^^^

^a^ Adjusted marginal probabilities were estimated from fully adjusted multilevel logistic models with random intercepts for health systems and patients. For hydromorphone and methadone, uncertainty is reported as 95% credible intervals (CrIs) from Bayesian multilevel logistic models; all other outcomes report 95% confidence intervals (CIs) from frequentist GLMMs. Calendar year was modeled as a factor. Drug-specific columns represent separate outcomes, indicating whether the specified drug was ordered during the encounter (Y/N) (outcomes are not mutually exclusive). ^b^ Excluded 308 records with missing sex information. * *p* < 0.05; ** *p* < 0.01; ^^^ 95% credible interval (CrI) excludes 1.0, indicating strong posterior evidence that the aOR differs from 2014. ^c^ Adjusted odds ratios (aORs) compare each calendar year with 2014 (reference) within each outcome column and were estimated from the same fully adjusted multilevel logistic models.

**Table 4:** Comparison of proposed framework with current approaches.

Existing Framework/Checklist	Core Area	Shared Elements in Our Framework	What We Reorganize/Operationalize	What Is New in Our Framework
**RECORD**	Transparency.	Cohort definitions, data selection transparency, and structural bias disclosure.	Transforming passive disclosure and reporting prior to publication into dynamic real-time software pipeline gates.	*Prospective runtime code* *validation;* checks the actual code against structural guidelines before model finalization.
**STaRT-RWE/HARPER**	Structured design templates and study protocol harmonization.	Aim/estimand specification, causal diagram mapping, and pre-planned sensitivity checks.	Converting design protocol text into programmatic requirements mapped across 10 functional coding domains.	*Closed-loop LLM semantic auditing*—the AI actively evaluates whether the script matches the planned estimand design.
**Jaksa et al. Framework**	Stakeholder trust, fitness-for-use, and data quality governance.	Data quality check techniques, transparent variable coding, and baseline diagnostics.	Operationalizing high-level trust criteria into clear, sequential validation stages (specification, modeling, and robustness).	*Hierarchical validation* *matrices*—links the restricted LLM prompt and routs automatically to trap unexamined data anomalies.

## Data Availability

The data used in this study were obtained from Cerner Corporation (North Kansas City, Missouri) through a data licensing agreement with the Real-World Data Institute and are not publicly available. Researchers interested in accessing these data should contact Cerner Corporation directly regarding licensing and availability.

## Supplementary Materials

The following supporting information can be downloaded at: https://www.mdpi.com/article/10.3390/bioengineering13101098/s1, Figure S1: After removal of ‘as needed’ orders, examination in GLMM procedure of availability of complete dosage information (Y/N) for exposure calculation and the proportional contribution of random effects in the hierarchical data structure: variation attributable to health systems, patients within health systems, and encounters within patients; Table S1: Apply exclusion criteria to identify eligible prescription rows for analyses; Table S2: Missing rate for variables important to assess daily dose (mg/kg) and MME for opioids across valid orders 1 January 2014–31 December 2021; Table S3: Analysis of potential omissions in drug names lacking active ingredients (Trade Names) based on a random sample of 10,000 claims for each active ingredient in Cerner RWD among patients 0–21 years of age and hospitalized from 2014–2021; Table S4: Proportion of variability attributable to each hierarchical level for outcome of an opioid being ordered during patient’s hospitalization (Y/N): comparison of base model, model adjusted for systems-level variables, case-mix of patients, and the fully adjusted model using the GLMM framework.
