# Supplementary material for: A Bias-Aware Analytic Framework for Real-World Data with AI-Audited Workflows: Application to Opioid Ordering in Pediatric Oncology
Source: Bioengineering (Basel). 2026 Sep 23;13(10):1098. doi: 10.3390/bioengineering13101098 (PMC13702015; doi:10.3390/bioengineering13101098)
Supplement: Supplementary file 1 [file bioengineering-13-01098-s001.zip › Tables S1–S4.pdf]

Supplemental Table S1. Apply exclusion criteria to identify eligible prescription rows for analyses.

		Exclusion criteria applied in sequential order, n:				
		Step 1 StartDate before Admit Date	***Step 2 Status*** = Cancelled or 'Error entry deleted'	***Step 3 Drug*** combinations w/other active ingredient(s) [a]	***Step 4 As Needed*** = True	
**Active Ingredient**	**Total No. Rx rows for encounters N**	n (row %)	n (row %)	n (row %)	n (row %)	**Final No. Rx rows for analyses N**
**Morphine**	81,733	566 (0.7%)	861 (1.1%)	1,976 (2.4%)	34,490 (42.2%)	43,840
**Fentanyl**	58,182	359 (0.6%)	265 (0.5%)	2,098 (3.6%)	11,411 (19.6%)	44,049
**Oxycodone**	50,057	346 (0.7%)	508 (1.0%)	724 (1.4%)	33,568 (67.1%)	14,911
**Hydromorphone**	19,847	118 (0.6%)	183 (0.9%)	24 (0.1%)	6,927 (34.9%)	12,595
**Methadone**	8,441	10 (0.1%)	331 (3.9%)	0 (0.0%)	240 (2.8%)	7,860
**Total**	218,260	1,399 (0.6%)	2,148 (1.0%)	4,822 (2.2%)	86,636 (39.7%)	123,255
# Remaining	***218,260***	*216,861*	*214,713*	*209,891*	*123,255*	***123,255***

[a] Drug combination excluded: Morphine: *Nalbuphine, Butorphanol, Buprenorphine, and Oxymorphone*; Fentanyl*: Fentanyl-Bupivacaine, Fentanyl-Ropivacain, and Remifentanil*; Oxycodone: *Acetaminophen-Oxycodone and Ibuprofen-Oxycodone;* hydromorphone*: bupivacaine-hydromorphone*.

[b] As needed outcome: order excluded due to 'as needed'=TRUE, among the 215,839 remaining after step 3 (Y/N);

[c] Final outcome: order excluded from final analyses set, among the 224,243 originally considered (Y/N); models excluded one tenant with no variation in outcome (tenant 50 had two orders and both were indicated as needed).

Supplemental Table S2. Missing rate for variables important to assess daily dose (mg/kg) and MME for opioids across valid orders Jan 1, 2014 – Dec 31, 2021.

		MISSING RATE, n (row%)			
**Active Ingredient**	**Final No. Rx rows for analyses**	**Frequency**	**Dose Quantity**	**Route**	**Complete Dose Data** [a]
**Morphine**	**43,840**	6,170 (14.1%)	20,030 (45.7%)	18,196 (41.5%)	**12,853 (29.3%)**
**Fentanyl**	**44,049**	5,999 (13.6%)	14,988 (34.0%)	15,999 (36.3%)	**18,009 (40.9%)**
**Oxycodone**	**14,911**	1,342 (9.0%)	6,696 (44.9%)	8,199 (55.0%)	**5,284 (35.4%)**
**Hydromorphone**	**12,595**	3,525 (28.0%)	4,578 (36.3%)	6,831 (54.2%)	**2,960 (23.5%)**
**Methadone**	**7,860**	484 (6.2%)	2,463 (31.3%)	3,122 (39.7%)	**4,029 (51.3%)**
**Total**	**123,255**	17,520 (14.2%)	48,755 (39.6%)	52,347 (42.5%)	**43,135 (35.0%)**

[a] Range across years: 29.2% - 37.8%; health systems range after restricting to those with >=5 orders: 3.8%-100% (mean=49.2%, SD=24.5)

Supplemental Table S3. Analysis of potential omissions in drug names lacking active ingredients (Trade Names) based on a random sample of 10,000 claims for each active ingredient in Cerner RWD among patients 0-21 years of age and hospitalized from 2014-2021. Methadone did not have trade names listed.

**Morphine**<br>**N=10,000**	**Fentanyl**<br>**N=10,000**	**Hydromorphone**<br>**N=10,000**	**Oxycodone**<br>**N=10,000**
**Generic: 9,992 (~100%)**<br><br>**Omissions: 8 (<0.1%)**<br><br>**<u>Trade Names Checked</u>**<br>n=0, Arymo ER [b]<br>n=1, Astramorph-PF [a]<br>n=0, Avinza [b]<br>n=0, DepoDur [b]<br>n=0, Duramorph [a, b]<br>n=0, Infumorph [a]<br>n=1, Kadian [a, b]<br>n=0, Morphabond [b]<br>n=1, Morphabond ER [a]<br>n=5, MS Contin [a]<br>n=0, Paregoric [b]<br>n=0, Roxanol [b]<br>n=0, Statex [b]	**Generic: 9868 (98.7%)**<br><br>**Omissions: 132 (1.3%)**<br><br>**<u>Trade Names Checked</u>**<br>n=0, Abstral [a, b]<br>n=0, Actiq [a,b]<br>n=1, Duragesic [a, b]<br>n=0, Fentora [a,b]<br>n=0, Ionsys [b]<br>n=0, Lazanda [a]<br>n=0, Onsolis [b]<br>n=132, Sublimaze [a]<br>n=0, Subsys [a]	**Generic: 7,677 (76.7%)**<br><br>**Omissions: 2,323 (23.2%)**<br><br>**<u>Trade Names Checked</u>**<br>n=2323, Dilaudid [a, b]<br>n=0, Dilaudid-HP [b]<br>n=0, Exalgo [a, b]<br>n=0, Palladone [b]	**Generic: 9,779 (97.8%)**<br><br>**Omissions: 221 (2.2%)**<br><br>**<u>Trade Names Checked</u>**<br>n=7, Oxaydo [a, b]<br>n=0, Oxecta [b]<br>n=49, Oxycontin [a, b]<br>n=0, Oxy IR [a]<br>n=164, Roxicodone [a, b]<br>n=0, RoxyBond [b]<br>n=0, Xtampza [a]<br>n=1, Xtampza ER [b]

Generic would be included in final sample as implies opioid name was listed in drug name description.
[a] Olsen, Y. & Sharfstein, J. M. (2021). The Opioid Epidemic: What everyone needs to know. Oxford University Press. (Appendix 4).[32]
[b] Source: Modern Recovery Network Link. https://www.modernrecoverynetwork.com/opioid-prescription-addiction-generic-opioid-prescriptions/ . Accessed 2024 April 4.[33]
Note. Both references cite the USA Food and Drug Administration. Drugs@FDA database (source [a] sites accessing in 2018; source [b] sites accessing FDA: https://www.fda.gov/drugs/development-approval-process-drugs/drug-approvals-and-databases as of last access to source [b] on April 3 2024).[34]

Supplemental Table S4. Proportion of variability attributable to each hierarchical level for outcome of an opioid being ordered during patient's hospitalization (Y/N): comparison of base model, model adjusted for systems-level variables, case-mix of patients, and the fully adjusted model using the GLMM framework.

Variance Components	M1:Base model (unadjusted)	M2:Systems Level Factors [a]	M3:Case-Mix Factors [b]	M4:Forced "Fully" Adjusted	Interpretation in each model
					Variance attributable to differences:
$\sigma^2_{\text{health systems}}$	0.224	0.104	0.082	0.084	between health systems
$\sigma^2_{\text{health systems:patients}}$	0.919	0.919	0.839	0.837	across patients within the same heath system
$\sigma^2_{\text{health systems:patients:encounters}}$	1.140E-12	0.000	7.278E-13	8.847E-13	across encounters within the same patient
$\sigma^2_{\text{residual}}$ [c]	3.290	3.290	3.290	3.290	Residual (unexplained) variance [c]
					Proportion of total variance explained by:
**ICC** $_{\text{health systems}}$	0.050	0.024	0.019	0.019	clustering at the health system level, reflecting differences between health systems.
**ICC** $_{\text{health systems:patients}}$	0.207	0.213	0.199	0.199	clustering at the patient level, reflecting differences between patients within the same hospital.
**ICC** $_{\text{health systems:patients:encounters}}$	2.572E-13	0.000	1.729E-13	2.101E-13	differences between encounters within the same patient, after accounting for variability at the patient and health system levels.
**ICC** $_{\text{Total}}$	0.258	0.237	0.219	0.219	all levels of clustering in the hierarchical structure, including health systems, patients within health systems, and encounters within patients.
**LR-test**	Reference	$\chi^2_{(11)}$=25.689 P=.007	$\chi^2_{(37)}$=2741 P<.001	$\chi^2_{(44)}$=2762 P<.001	
**AIC**	92347	92344	89680	89673	Lowest AIC is considered the best-fitting model, as it achieves the optimal balance between goodness-of-fit and complexity (parsimonious). Predictive value requires additional diagnostic checks of residuals.

[a] Systems level factors: Health system volume for eligible encounters (low: <=4 per year , moderate: 5-222 per year , high: >222 per year), ZIP based on mode of patient ZIP first digit within health system (9 U.S. Regions), and year of admit (2014-2021).

[b] Case-mix factors: age (0-1, 2-12, 13-17, 18-21), sex (M/F), payor (public, private, other/unknown), LOS (0, 1, >=2), and as described in Table 1: ethnicity/race (8 categories), admit type (5 categories), and initial diagnoses (8 categories).

[c] Baseline variance component of the logistic distribution is assumed fixed and estimated at $3.29=\pi^2/3=\sigma^2_{\text{fixed baseline variance}}$)

$\sigma^2_{\text{Total}} = (\sigma^2_{\text{health system}} + \sigma^2_{\text{healthsystem:patient}} + \sigma^2_{\text{healthsystem:patient:encounter}} + \sigma^2_{\text{fixed baseline variance}})$

$\text{ICC}_{\text{Health System}} = \sigma^2_{\text{health system}}/\sigma^2_{\text{Total}}$; $\text{ICC}_{\text{Health System:Patient}} = \sigma^2_{\text{health system:patient}}/\sigma^2_{\text{Total}}$; $\text{ICC}_{\text{health system:patient:encounter}} = \sigma^2_{\text{health system:patient:encounter}}/\sigma^2_{\text{Total}}$

$\text{ICC}_{\text{Total}} = (\sigma^2_{\text{health system}} + \sigma^2_{\text{healthsystem:patient}} + \sigma^2_{\text{healthsystem:patient:encounter}})/\sigma^2_{\text{Total}}$
